# Moral Uncanny Valley revisited – how human expectations of robot morality based on robot appearance moderate the perceived morality of robot decisions in high conflict moral dilemmas

**DOI:** 10.3389/fpsyg.2023.1270371

**Published:** 2023-11-21

**Authors:** Michael Laakasuo

**Affiliations:** ^1^Faculty of Medicine, Department of Psychology and Logopedics, University of Helsinki, Helsinki, Finland; ^2^Faculty of Social Sciences, Department of Social Research, University of Turku, Turku, Finland

**Keywords:** moral judgment, Moral Uncanny Valley, moral psychology of AI, AI, robotics, uncanny, Uncanny Valley

## Abstract

In recent years a new sub-field of moral psychology has emerged: moral psychology of AI and robotics. In this field there are several outstanding questions on how robot appearance and other perceived properties of the robots influences the way their decisions are evaluated. Researchers have observed that robot decision are not treated identically to human decisions, even if their antecedents and consequences are identical to human decisions. To study this moral judgment asymmetry effect further, two studies with a series of high conflict moral dilemmas were conducted: Study 1 – which used photorealistic full body imagery -- revealed that utilitarian decisions by human or non-creepy (i.e., nice) looking robotic agents were less condemned than “creepy” (i.e., unease inducing) robots, whereas “creepy” robots received higher moral approval when making deontological decisions. Furthermore, an exploratory analysis demonstrated that the creepiest robot did not cause moral surprise or disappointment when making utilitarian decisions. However, Study 2 showed that mere symbolic representation of the agent’s face did not trigger the Moral Uncanny Valley (where decisions of creepy robots are perceived negatively), suggesting that the effect is dependent on the photorealistic appearance of the agent. These results are in tension with some previous findings in robot moral judgment literature. Future research should focus on creating standardized stimuli for studying moral decisions involving robots and elucidating the complex interactions between agent appearance, decision type, and pre-decision expectations. This work deepens our understanding of the relationship between a decision-making agent’s appearance and the moral judgment of their decisions. The findings have significant implications for the design and implementation of autonomous agents in morally charged situations.

## Introduction

In the very near future, humans will be working even more closely with different types of AI assistants, whether embodied in robots or other ubiquitous or distributed systems such as smart homes or self-driving cars. Technologies, similar to ChatGPT, will be integrated with these localized and distributed artificial agents, with whom humans will be engaging in joint work, decision-making, and various sorts of collaborations (Savela et al., 2018, 2021).

However, since humans evolved in environments where moral decisions were mostly made by other humans, it feels unintuitive for humans to evaluate the moral decisions of machines (e.g., Malle et al., 2016, 2019). The basic evolutionary mismatch-hypothesis suggests that humans will not be able to evaluate modern autonomous technologies that are based on probability calculus and symbolic logic, intuitively or morally appropriately (Laakasuo et al., 2021a,b,c). However, we are capable of evaluating moral decisions made by other humans, and doing so deeply activates our social cognition (Voiklis and Malle, 2017). When humans interact with social robots, our norm and value perception systems are activated (Voiklis and Malle, 2017; Bigman and Gray, 2018; Stuart and Kneer, 2021).

We will now review some literature on how this evolutionary mismatch-hypothesis manifest itself in the current literature on moral judgments of robot decision-making. We will then summarize the current state-of-the-art regarding the uncanny valley effect and then we will look at how these topics interact. We are looking to understand how the robot’s appearance and human perception of it, influences the moral judgments of its decisions.

### Asymmetries in moral judgments of human vs. robot decisions

Unfortunately, the explanation that humans do not perceive machines as appropriate decision-makers is made more nuanced by recent moral psychological research that seems to have uncovered a phenomenon that still does not have a proper name, but here we will call it the “asymmetry effect.” In other words, humans are not overall averse to machines making decisions (e.g., Bigman and Gray, 2018), but only to certain decisions made by those machines (see Laakasuo et al., 2023; Sundvall et al., 2023). For instance, we allow humans to make forced medication decisions, but not machines, even if the antecedents and the consequences of the decisions are identical in both cases (Laakasuo et al., 2023); nonetheless, we allow both human and machine nurses to disobey the orders given to them when those orders might violate the patient’s autonomy. If there was a general aversion to machines making decisions, it should be equally distributed to all kinds of decisions that the machines make, but this is not the case.

In marine rescue situations, where an emergency is caused by two drunk individuals, humans morally judge the rescue robot’s decision as bad if it saves those who caused the accident over a single innocent victim (Sundvall et al., 2023). However, human lifeguards are allowed to make whichever decision they want, and their decision is not judged more harshly as a consequence (Sundvall et al., 2023). This is especially striking, since the utilitarian option of saving the most lives is only allowed for a human, not for a robot. Nevertheless, Sundvall et al. (2023) also showed that the moral judgments of the agent’s decisions were not just based on their perceived mental capabilities, but also on their appearance and their presumed bodily shapes (see also: Laakasuo et al., 2021d). This further questions the “mind perception” hypothesis as a single explanation of this phenomenon, because the current literature suggests that these differences or “asymmetries” appear only with certain decisions and are modulated by the perceived appearance of the decision-maker in some cases (Malle et al., 2015, 2016, 2019).^1^

The asymmetry effect was first observed in a study of the traditional trolley problem. Malle et al. (2015) found that individuals were held more accountable for a utilitarian action (sacrificing one life to protect five) compared to a deontological approach (taking no action, thereby resulting in the loss of five lives). However, regardless of the choice made, the level of blame attributed to a robot was the same and did not exceed that attributed to humans. This implies that people judge humans based on the choices they make, but robots are blamed regardless of the choices they make. Furthermore, when examining the moral severity of actions (rather than attributing blame), a robot was judged more harshly for choosing the deontological course, whereas the human was judged more severely when they went for the utilitarian option (Malle et al., 2015). This finding was replicated by Komatsu et al. (2021), who showed that Japanese participants evaluated a robot more negatively than a human when they chose the omission (deontological, inaction) option.

Similarly, Malle et al. (2019) studied moral judgments of military decisions. The participants were given a description where either a human pilot or an AI was ordered to launch a missile strike that could harm innocent bystanders. Participants attributed more blame to the human when they decided to disobey the order compared to when they followed the order. Conversely, the AI was blamed equally for both decisions. Interestingly, the AI was attributed with less blame for disobeying the order than the human pilot. In other words, the participants viewed the AI’s decision to disobey more positively compared to the same decision made by a human.

To summarize, the human-robot moral judgment asymmetry effect has been observed in traditional trolley dilemmas, rescue situations, forced medication decisions and military contexts. However, this literature has not really taken into consideration the expansive literature on robot appearance. This is an important detail, since other factors may play an important role in moral judgments of AI decisions, such as how creepy or uncanny they seem to humans, which we will look at next.

### Uncanny Valley effect

**Figure 1:**
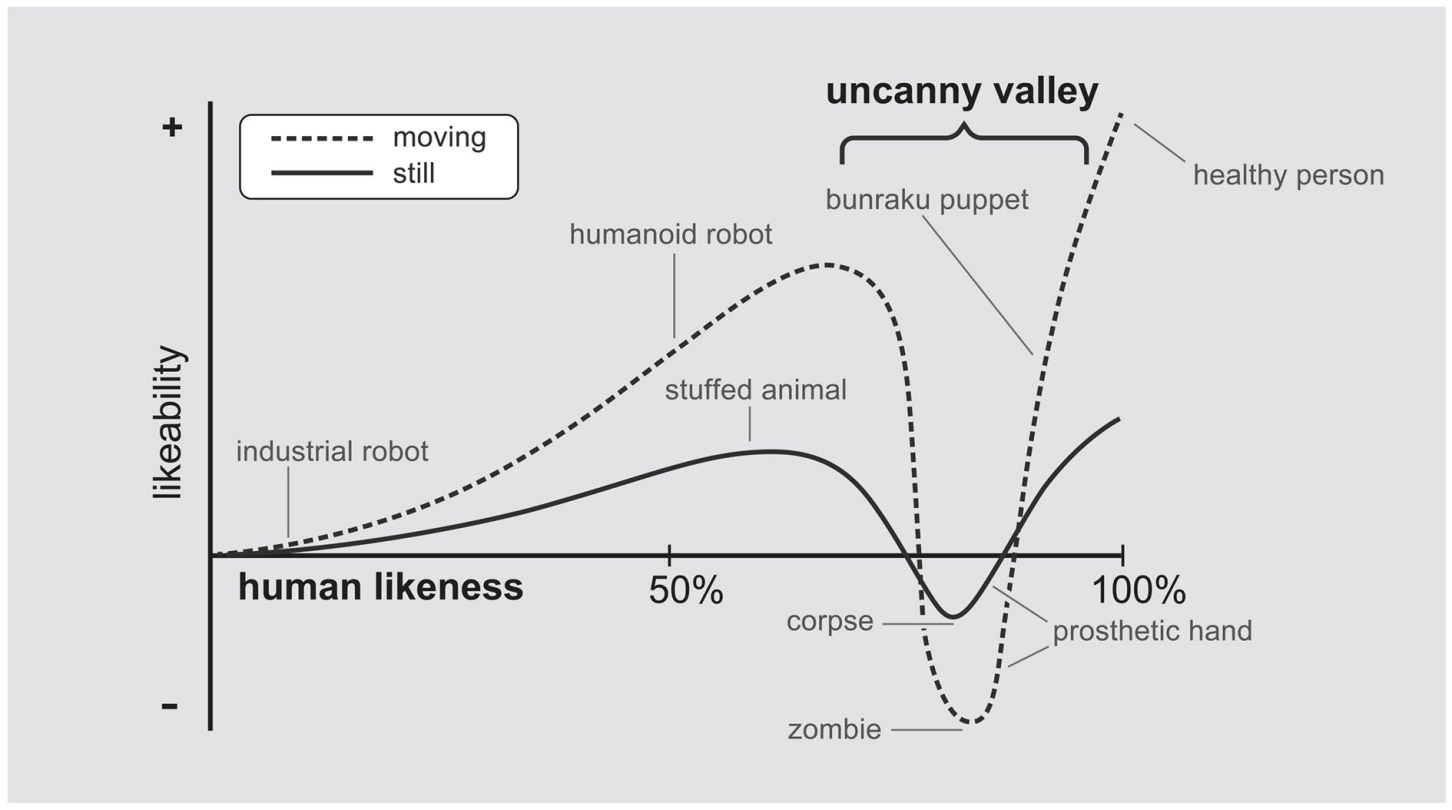
Mori (1970) suggestion for the Uncanny Valley phenomenon. Picture version adapted from Wikipedia and modified by us to suit the context of this paper. Note that this valley shape is a third degree polynomial function.

Recently, researchers have identified the Uncanny Valley phenomenon: when robots resemble humans too closely, but not perfectly, they are considered “creepy” and uncomfortable (see Figure 1; Palomäki et al., 2018; Diel et al., 2021). However, if the robots clearly look like robots or do not incite creepiness, they are favored. Most research on the Uncanny Valley Effect (UVE) has focused on boundary conditions and the replicability of the phenomenon using various stimuli (see Diel et al., 2021). According to a recent review, there is no consensus on a scientific explanation for this phenomenon (Diel et al., 2021). Also, the implications of the Uncanny Valley Effect are yet to be thoroughly examined outside the “boundary condition” paradigm. Information varies depending on the conditions and the dependent variables under which the phenomenon emerges and on the robustness of the phenomenon (Diel et al., 2021). Despite suggestions to study UVE under conditions other than just basic research, only a few papers have emerged, and only one focuses on the moral psychological implications of UVE (Laakasuo et al., 2021d). Other studies have examined human-like vocal mimicry (Aylett et al., 2019; Kirkby et al., 2023), ways to improve human perception of robots in challenging situations (Grundke et al., 2023), or methods to decrease or lessen the severity of the UVE.

Given the challenges in replicating the UVE phenomenon under different conditions, this is not surprising. However, since it clearly exists, the most robust ways of replicating it could provide insights into the phenomenon and its reach beyond the boundary conditions paradigm. For instance, it would be beneficial to know whether the level of uncanniness of a digital assistant would influence driving instructions, but would not impact advice on investments or moral decisions. Such information could help us understand why it exists to begin with, and if it is activated in certain situations (but not others), it could point to potential cognitive mechanisms behind it. This paper is a small step in that direction.

Indeed, recent research on the Uncanny Valley has expanded toward moral psychology (Laakasuo et al., 2021d), and it is not clear whether the “Moral Uncanny Valley” effect extends beyond a single set of stimuli. As we know from previous studies in human-robot interaction, a robot’s appearance influences how people react to their actions and decisions, but which aspects of the robots appearance, that is not clear. We will now look at some research on how robot appearance and human perceptions of robots moderates human reactions toward them.

### Robot appearance affects human reactions toward them

In their review of anthropomorphism in robots, Złotowski et al. (2015) argued that attributing “humanness” to machines depends on their perceived human-like appearance and behavior (particularly when interacting with humans). The robot’s perceived fluency in communication and non-verbal gestures, along with the capacity for emotion and intelligence, are factors that may enable humans to view robots as “human.” Appearance also plays a significant role. For instance, Trovato et al. (2018) concluded that minor modifications to the humanoid appearance of robots affect how humans project mental properties and capabilities onto them. Similarly, Addison et al. (2019) demonstrated that merely changing a robot’s color alters people’s implicit associations with its mental properties. The Uncanny Valley literature also suggests these associations may not always be linear (Palomäki et al., 2018), making the task of evaluating people’s reactions to robots and their appearances challenging. This difficulty is further exacerbated by a lack of stimulus material capable of independent replications (see Laakasuo et al., 2021d, for discussion).

In related research, Yogeeswaran et al. (2016) showed participants mock video interviews with a very human-like and non-human-like robot. The video narrator stated that the robot either had various mental and physical capacities, or that its capacities supersede humans in these tasks. Participants only perceived the human-like robot as a threat if it outperformed them. In a similar study, Złotowski et al. (2017) presented participants with various types of robots. The narration suggested that the robot could either make autonomous decisions, or merely follow instructions from humans. Here too, the robots were perceived as more threatening when described as autonomous.

Some researchers speculate that as artificial agents become more human-like, the likelihood of treating them as moral agents increases (Bigman et al., 2019). There is some evidence supporting this notion. For example, Syrdal et al. (2008) showed that when robots resemble humans more closely, they are expected to adhere to human notions of personal space more accurately. Kwon et al. (2016) found that more human-like robots are expected to empathize with human emotions. In the realm of moral psychology, Malle et al. (2016) showed that non-humanoid robots were perceived as more blameworthy for deontological decisions than utilitarian ones.

Interestingly, this contrasts slightly Laakasuo et al. (2021d), who used variations of the trolley dilemma and found that decisions of more human-like robots, whether deontological or utilitarian, were considered less moral. This discrepancy could be due to the Uncanny Valley effect’s reliance on photorealistic anchors, which are more prevalent in facial stimuli rather than full-body stimuli (Palomäki et al., 2018; Diel et al., 2021). However, there is another crucial difference between Malle et al. (2016) and Laakasuo et al. (2021d). Malle et al. (2016) used a low stakes moral dilemma (Switch version of the trolley dilemma), and Laakasuo et al. (2021d) used high conflict moral dilemmas (Footbridge version and its variations; see Laakasuo and Sundvall, 2016). Indeed, a recent study by Zhang et al. (2022) showed that there is a larger expectation for robots to be utilitarian in high-conflict moral dilemmas compared to impersonal dilemmas as used by Malle et al. (2015, 2016).

### Current studies

In summary, it appears that people attribute human properties to artificial agents based on their appearance. Depending on contextual factors, such as their own expectations and experiences (see Koverola et al., 2022b), humans anthropomorphize artificial agents differently. Some robots seem more threatening than others (Palomäki et al., 2018), while the Uncanny Valley Effect plays an influential role in these perceptions. Furthermore, we are not fully aware of how a robot’s position on the human-likeness continuum affects our moral judgments of their decisions (Malle et al., 2016; Laakasuo et al., 2021d). Yet, we know that there is a consistent asymmetry in how different decisions made by either humans or robots are evaluated, and this effect is probably reversed or larger in high-conflict dilemmas, which we also use here (Zhang et al., 2022). This research is motivated by a recent statement made by Sullivan and Wamba (2022), stating that scientists need to consider the AI agent’s appearance in future research on moral evaluations toward them. This is our aim here. Since this type of research is in its infancy, we will start with basic high conflict trolley-type dilemmas, which have become a standard trope in moral psychological literature (Christensen and Gomila, 2012; Laakasuo and Sundvall, 2016; Laakasuo et al., 2017; Greene et al., 2008; Zhang et al., 2022).

Thus, in this article:

- We examine whether robot decisions are evaluated differently from human decisions, as a function of the robot’s appearance, if the stimulus material conforms to the Uncanny Valley (Study 1). In a previous Moral Uncanny Valley paper by Laakasuo et al. (2021d), the stimulus was focused on robots’ faces; here, we focus on the full body of the robot and we use pretested stimulus material by Palomäki et al. (2018)
- We investigate whether the human-robot asymmetry effect occurs in certain cases (omission vs. commission) and whether it is moderated by the robot’s appearance in high conflict dilemmas (Study 1). Previous research did not report any differences in the type of decision made, and there appears to be a discrepancy between Malle et al. (2016) and Laakasuo et al. (2021d). We aim to further investigate this issue. Based on Zhang et al. (2022), we expect our pattern to be reversed to what Malle et al. (2016) report, namely that robots are judged more harshly for deontological/omission decisions.
- We add the perceived creepiness and level of perceived mind of the target as a covariate in our model (Study 1), since the Uncanny Valley Effect is reported to result from the perceived creepiness of the agent.
- We also demonstrate with a pretested stimulus that the Moral Uncanny Valley Effect does not appear when the stimulus material does not adhere to certain standards (Study 2).

## Study 1 – when stimulus material aligns with the Uncanny Valley

The aim of this study was to investigate the potential replicability of the previously reported Moral Uncanny Valley effect (Laakasuo et al., 2021d) with different stimulus materials. Previously, Laakasuo et al. (2021d) used four agents’ faces, while here we used whole body images (adopted from Palomäki et al., 2018; Studies 3A and 3B). The study was preregistered.^2^

### Method

#### Participants

Our aim was to collect 700 participants, 70 per cell. We collected the data through Prolific Academic^3^ and we excluded participants who failed the attention check (*n* = 28). Participants were also filtered (on prolific) for not having any ongoing health issues, for having previously completed at least 50 surveys successfully and for fluent English skills (*n* = 655; also inspected after data-collection). Two participants were excluded for taking suspiciously long (over 90 min). Our final sample size was 624 participants (261 = male; *M*age = 36.55; *Sd*age = 12.80); one person did not report their gender. Participants were compensated according to Prolific Academic rules.

#### Procedure and design

**Figure 2:**
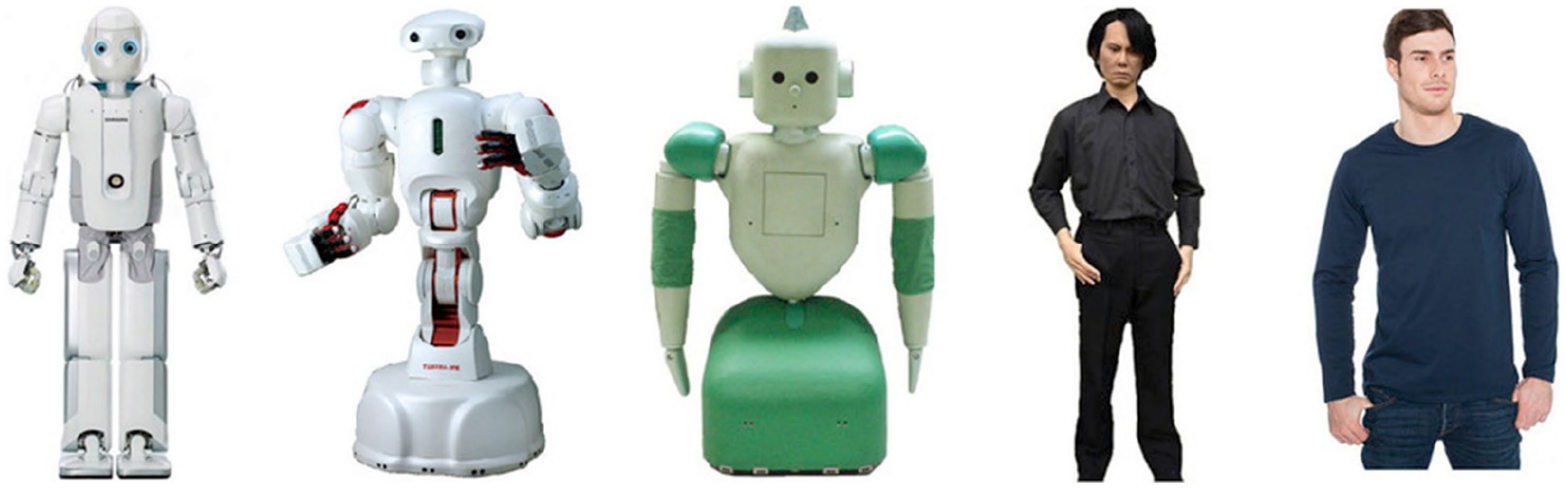
Pictures of the agents used in Study 1. From left to right: Robot 1, Robot 2, Robot 3, Robot 4, and Human.

After informed consent, participants were randomized into one of 10 conditions in a 2 [Decision: Deontological, Utilitarian] × 5 [Agent: Robot 1, Robot 2, Robot 3; Robot 4 and Human; see Figure 2] between-subjects design. Robots 3 and 4; were positioned at the bottom of the Uncanny Valley (based on Palomäki et al., 2018); correspondingly, Human and Robot 2 were positioned at the sides of the valley. This is in line with the general shape of the Uncanny Valley,. The images for the five different agents are depicted in Figure 2 (adopted from Palomäki et al., 2018).

First, participants were randomly shown a picture of an agent and requested to fill in the Mind Perception scale with respect to that agent. Next, participants were also requested to evaluate the level of perceived baseline morality, creepiness, trustworthiness and likability of the agent (see Manipulation checks below). Then, participants completed the actual task (see Dependent Variable), as well as additional exploratory measures (reported elsewhere). The survey ended with the assessment of standard demographic information and a debriefing.

### Materials

#### Perceived baseline morality of the agent

**Figure 3:**
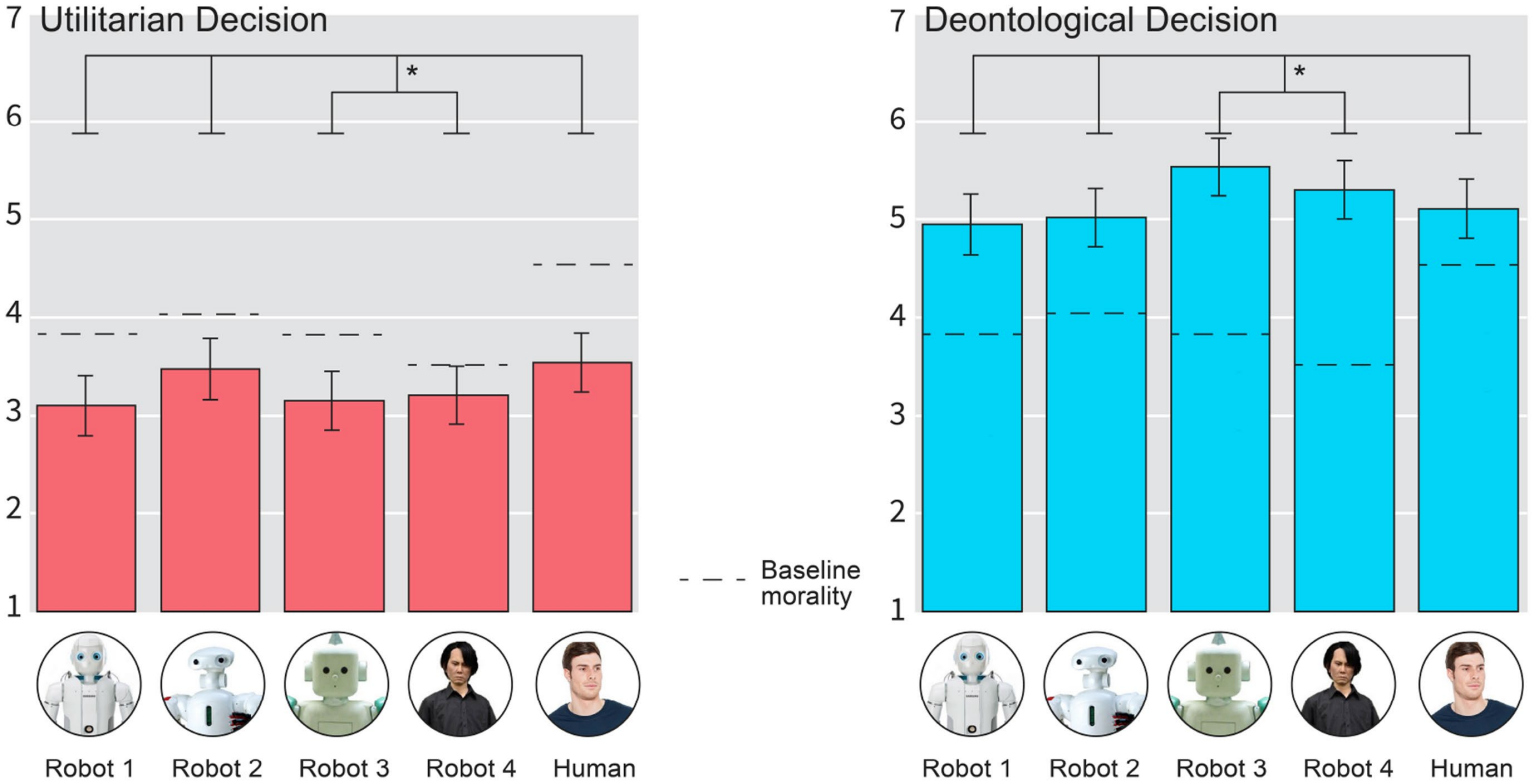
The perceived morality of the decision made by all agents. The black line is the baseline morality measured before the decision evaluation tasks. Error bars are 95% CIs. High resolution image, please zoom in.

For exploratory purposes, we included a measure of perceived baseline morality of the agent. This question was formulated as” How moral do you think the agent you see in the picture is?”; and was anchored from 1 (Not at all) to 7 (Very much). The level of perceived morality is shown in Figure 3 (Results section). The human agent is perceived to be the most moral agent, where as the least moral agent is considered to be Robot 4 (Ishiguro), and all other robots agents falling between the two.

##### Mind perception scale

**Figure 4:**
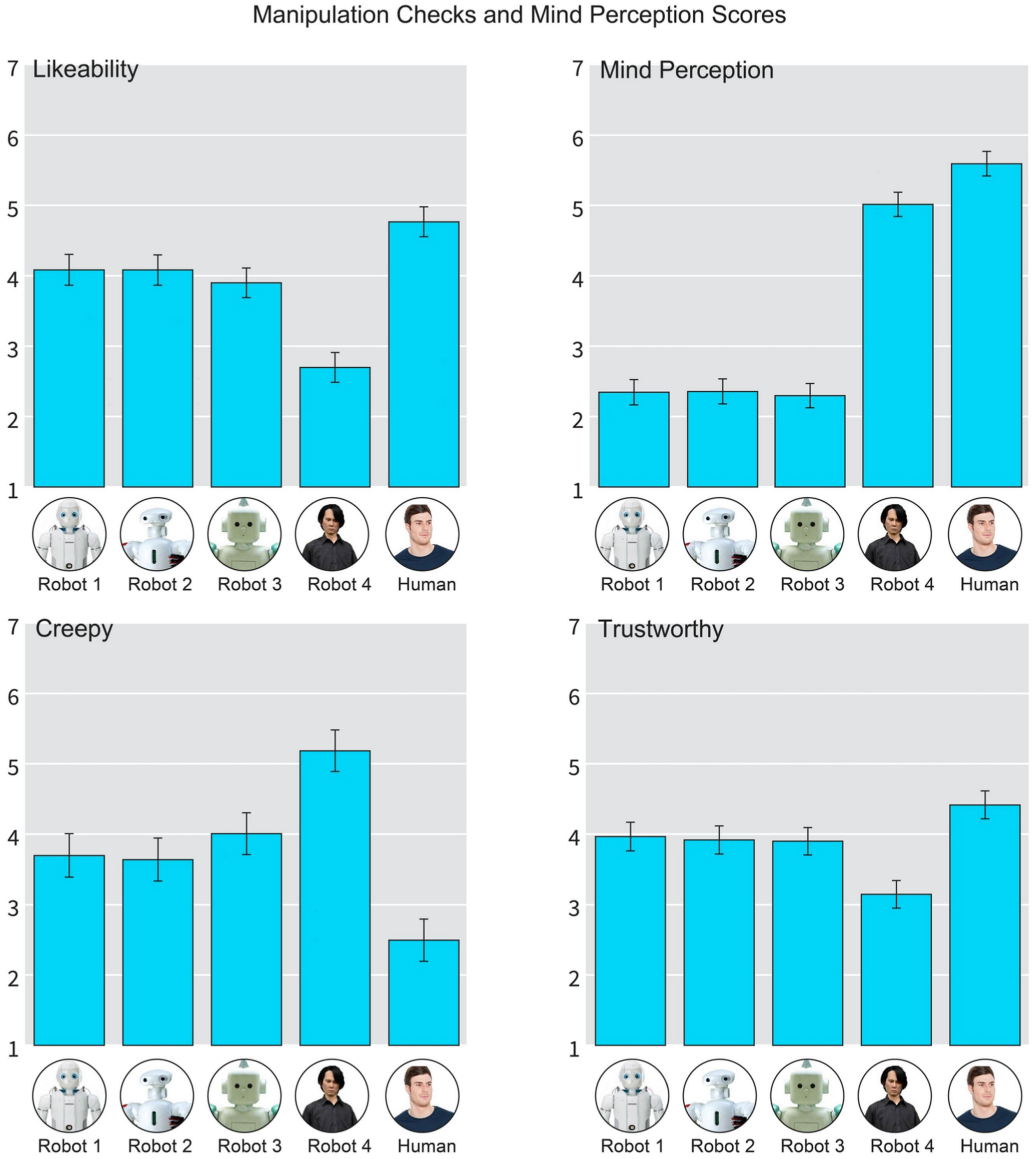
Summary graph of manipulation checks and mind perception attributed to the agent. Error bars are 95% CIs. High resolution image, please zoom in.

For exploratory and manipulation check purposes we employed the 17-item Mind Perception Scale by Gray and Wegner (2012). This scale measures how much mind and capabilities for experiences we assign to different objects, agents, humans and animals. The scale has 4 sub-scales, but for the purposes of this study we averaged all the items together, as all the items had very high Cronbach’s alpha (0.97). For most of the scale items the participants responded to the questions in the form “This agent on the left can X [e.g., feel pain].” For each of the four subscales [capability for (a) pain, (b) experience (c) agency, and (d) consciousness] higher scores indicate more perceived mental capabilities for the agent. Example item(s) for (a) pain: “X can experience pain.” (b) experience: “X can experience feelings,” (c) agency: “X can influence the outcome of situations” and (d) consciousness: “X is conscious of the people and the world around him/her/it.” The questions were anchored from 1 (Not at all) to 7 (Very much). For Mind Perception scores per agent, see Figure 4.

##### Dependent variable

Participants evaluated five trolley-dilemma type vignettes in random order, with the agent shown on the left side of the dilemma (*cf.* Supplementary Appendix). Between the vignettes, participants indicated how moral they found the agent’s decision to be, on a scale from 1 (Very Immoral) to 7 (Very Moral). In the preregistration we mention four dilemmas (Mountaineering, Terrorist, Submarine, Footbridge; see Greene et al., 2008, Laakasuo and Sundvall, 2016), which were the same as those mentioned in Laakasuo et al. (2021d); see Supplementary Appendix); however, here we also included a fifth dilemma (Euthanasia). We report the results for the four item DV under the heading Preregistered Analys and the Five Item version under Exploratory analyses. The results are, for all practical purposes, the same. For the four item version the Cronbach’s α = 0.83; for the 5 item version 0.85. All the measures were averaged together to form a perceived decision morality scale. Higher scores indicate higher perceived morality of the decision.

#### Manipulation checks

Since we used pretested stimulus materials from Palomäki et al. (2018), we wanted to make certain that our stimulus materials replicate previous findings on some basic level. Indeed, as reported by Palomäki et al. (2018), Robot 4 was found to be at the bottom of the Uncanny Valley, whether measured with creepiness, likability or trustworthiness; and this dip/tilt already starts with Robot 3. See Figure 4.

As the literature also suggest (Gray and Wegner, 2012), that the Uncanny Valley Effect is driven by the level of Perceived mind in the agent. We therefore computed a single mind perception score for all the Agents in the study (see Figure 4). What is noteworthy, is that the mind perception scores and the Uncanny Valley items do not overlap; the second creepiest robot (Robot 3) is creepier than Robots 1 and 2 and is on the same level with mind perception scores. The creepiest robot (Robot 4) is close in Mind Perception scores with the Human, but also statistically significantly lower.

### Results

#### Preregistered analysis (4-item DV)

We ran a full-factorial two-way ANOVA for our 4-item DV (averaged perceived morality of decision in high conflict moral dilemmas) by using our experimental variables, Decision and Agent, as predictors. The main effect of the decision type (Utilitarian vs. Deontological) was significant (*F* > 380, *p* < 0.001). The main effect for the Agents’ appearance was not significant (*F* < 1.62, *p* = 0.17). This was due to a significant interaction effect, where the deontological decisions created a mountain shaped effect and the utilitarian decisions created a valley shaped effect: [*F*(4,614) = 2.61, *p* = 0.03]. As we were interested in the valley shaped contrasts, the omnibus test is not meaningful; we thus ran a valley shaped contrasts (third degree polynomial contrast weights: 0.1, 1.5, −1.60, −1.80, 1.80) for both the Utilitarian and the Deontological decisions separately and found that for the Utilitarian decision the valley shape contrast was statistically significant (*B* = 1.09, 95% CI [0.04, 2.08], *F*(1,614) = 4.18, *p* = 0.04); as was the Deontological decision, but to the opposite direction (|*B*| = 1.16, 95% CI [0.12, 2.20], *F*(1,614) = 4.86, *p* = 0.02). See Figure 3 below for the 5-item DV, which is essentially the same for 4-item DV.

#### Exploratory analysis (5-item DV)

We also ran a full-factorial two-way ANOVA for our 5-item DV (averaged perceived morality of decision in high conflict moral dilemmas) by using our experimental variables, Decision and Agent, as predictors. The main effect of the decision type (Utilitarian vs. Deontological) was significant (*F* > 370, *p* < 0.001). The main effect for the Agents’ appearance was not significant (*F* < 1.35, *p* = 0.25). This was due to a significant interaction effect, where the deontological decisions created a mountain shaped effect and the utilitarian decisions created a valley shaped effect: [*F*(4,614) = 2.61, *p* = 0.02]. As we were interested in the valley shaped contrasts, the omnibus test is not meaningful; we thus ran valley shaped contrasts (third degree polynomial contrast weights: 0.1, 1.5, −1.60, −1.80, 1.80) for both the Utilitarian and the Deontological decisions separately and found that for the Utilitarian decision the valley shape contrast was statistically significant (*B* = 1.08, 95% CI [0.07, 2.09], *F*(1,614) = 4.41, *p* = 0.03); as was the Deontological decision, but to the opposite direction (|*B*| = 1.17, 95% CI [0.17, 2.17], *F*(1,614) = 5.31, *p* = 0.02). See Figure 3 below for results.

We also tested the significance of a simpler quadratic contrast, where we assigned equal weights for the edges and the bottom of the valley (1 for Robot 2 and Human and − 1 for Robot 3 and Robot 4). Here, too, we found that for the Utilitarian decision, there was a clear valley shaped effect (*B* = 0.65, 95% CI [0.05, 1.25], *F*(1, 614) = 4.58, *p* = 0.03) and for the Deontological decision a” mountain shaped” effect for the Deontological decision (*B* = 0.70, 95% CI [0.11, 1.29], *F*(1, 614) = 4.58 *p* = 0.02). As a final step, we also compared human utilitarian decisions to two creepy robot utilitarian decisions; this was also statistically significant (*B* = 0.36, 95% CI [0.00, 0.72], *F*(1, 614) = 3.78, *p* = 0.05).

The results indicate, that the appearance of the robot clearly plays a role (Figure 3). However, it is not clear, whether the level of uncanniness plays a direct role in the context of this study, since it is the second most uncanny robot (Robot 3), which seems to be driving the effect, as our participants expect Robot 3 in deontological/omission/non-act situation to be the most moral actor as well as the least moral actor in the Utilitarian/commission/act situation.

To analyze this issue further, we added the perceived creepiness of the agent as a covariate into the model and retested for the contrasts. This resulted in removal of the valley-shape contrasts from the Utilitarian decisions (*B* = 0.74, *F*(1,613) = 1.86, *p* = 0.17); but not from the Deontological decisions (|*B*| = 1.40, 95% CI [0.38, 2.43], *F*(1, 613) = 7.23, *p* < 0.01).^4^ We then added the Mind Perception Scale as a covariate into the model; this time there was no marked shift in the valley-shaped contrasts in either Utilitarian [*B* = 0.99, *F*(1,613) = 3.70, *p* = 0.05] or Deontological decisions [|*B*| = 1.27, *F*(1, 613) = 6.24, *p* = 0.01]; albeit the statistical significances were slightly weaker due to drop in power as the model was more complex. The results seem to suggest, along with the manipulation check analysis, that the effects of the Moral Uncanny Valley effect – and thus the human-robot moral judgment asymmetry effects – are not driven by mind perception scores. We also ran a contrast analysis comparing human vs. the two uncanny robots Utilitarian decision, which was statistically significant (B = 0.36, 95% CI [0.00, 0.72] *F*(1, 614) = 3.78, *p* = 0.05).

As a final exploratory analysis, we created a difference variable, by deducting decision scores from the perceived baseline morality scores of the agent (black line in Figure 3). This means that negative numbers indicate a larger moral approval of the decision per agent, compared to the their expected baseline. Positive numbers indicate a smaller moral approval for what was expected of the agent initially.

**Figure 5:**
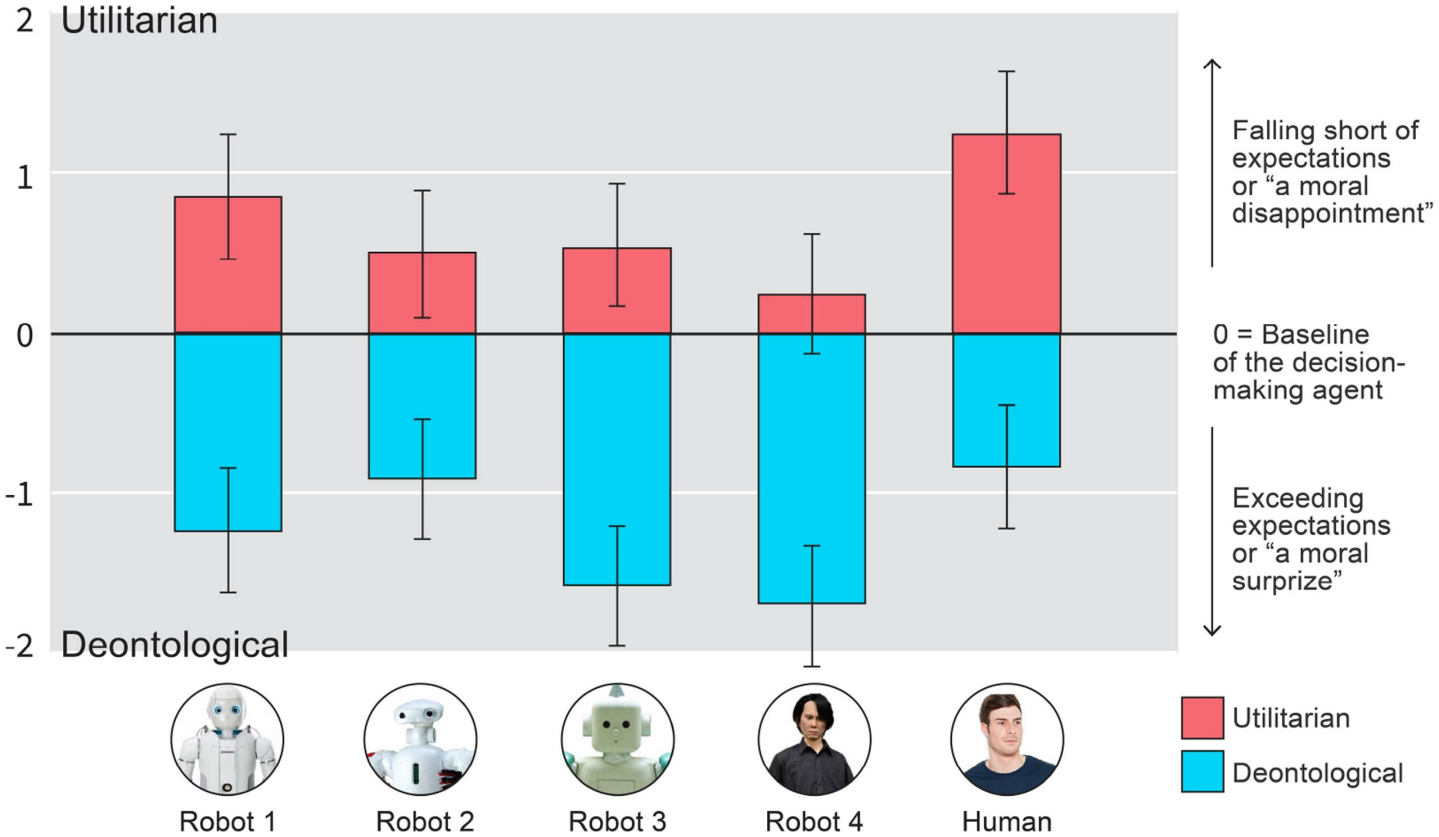
Higher scores indicate that the initial moral evaluations of the agents were more negative than the perceived morality of their decisions in High Conflict Moral Dilemmas. One could conceptualize negative scores as a positive moral surprise and positive scores as moral disappointment. Zero would be conceptualized as no difference between initial expectation and the morality of the decision that the agent made. Error-bars are 95% CIs.

We then ran a standard full-factorial ANOVA analysis by using the moral difference variable as the dependent variable and the by using our experimental variables as predictors, Decision and Agent, as predictors (see Figure 5 for results). This time, there were two main effects for both the Decision [*F*(1, 614) = 245.61, *p* < 0.001] and the Agent [*F*(4, 614) = 6.81, *p* < 0.001], but no interaction effect *F*(4, 614) = 1.18, *p* > 0.31. See Figure 5.

The results of the deviation score analysis seem to indicate that the two uncanny robots statistically significantly exceed their initial moral expectations more than the other agents when they decide not to act (deontological/omission decision): Valley-shaped contrasts for the deontological decision deviation score: (*B* = 2.60, 95% CI [1.33, 3.88], *F*(1, 614) = 16.09, *p* < 0.001).

This seems to imply, that the level of uncanniness of the agent matters for both: (a) moral expectations of their decisions and (b) to them as decision-makers. The results also seem to imply that there is largest” moral disappointment” when humans make utilitarian decisions in High Conflict Moral Dilemmas; in other words, although Human utilitarian decision is perceived as more moral than robot utilitarian decision (see above), it is in some sense the least expected (Valley shaped contrast analyses for the deviation scores in utilitarian decision: *B* = 1.77, 95% CI [0.48, 3.06], *F*(1, 614) = 7.29, *p* = 0.007; and Human vs. all the robots: *B* = 0.72, 95% CI [0.29, 1.14], *F*(1, 614) = 10.92, *p* < 0.001). Another interesting detail in the deviation score analysis is the fact that only the Ishiguro robot (Robot 4), when it made the Utilitarian Decisions, was not statistically significantly different from the Zero point (*B* = 0.025, 95% CI [−0.13, 0.68], *t*(70) = 1.32, *p* = 0.18); where as all the other agent-decision combinations were (|*Bs*| > 0.5, *|t*s| > 2.50 *p*s < 0.013). This indicates that when the most uncanny robot makes the utilitarian decision it is in line with the expectations projected onto it.

### Discussion of Study 1

It could be argued that the impact of the Uncanny Valley might be different when considering whole bodies versus faces. The Uncanny Valley Effect (UVE) is often associated with faces, and this shift to full bodies might have had an influence on the results, however these images have been shown to elicit the UVE in a similar manner to facial imagery (Palomäki et al., 2018). Furthermore, perceptions of what is “uncanny” could vary greatly among individuals and cultures, thus potentially influencing the results. For this reason, we also included the perceptions of robot likability in the analysis, and this did remove the effect from the Utilitarian decisions.

Future experiments can be strengthened by correlating the participants’ responses to their individual characteristics such as personality traits, attitudes toward robots, or previous experiences with robots. These factors could potentially influence how individuals react to “uncanny” robots and their decisions in moral dilemmas. We thank our reviewer for pointing these discussion points.

## Study 2 – when there is no Uncanny Valley

With this study we aimed to use a set of images produced by Ferrey et al. (2015) to induce the Uncanny Valley effect in order to study the possibility of the Moral Uncanny Valley in a laboratory environment. The stimulus material by Ferrey et al. (2015) hos not replicated its effects previously (Palomäki et al., 2018). Here we wish to see, whether a mere symbolic representation of robotic agents is enough to bring about the asymmetry effect mentioned in the introduction. If the effect surfaces in a material that does not induce the Uncanny Valley Effect, then the explanations for results of Study 1 would need to take this into consideration.

### Method

#### Participants

We set up a pop-up laboratory in a public library in Espoo, and recruited 221 participants (107 males, 114 females). The participants were, on average, 38.7 years old (*SD* = 16.8; range = 18–80). As the sample consisted of library users in the second biggest city in Finland it is much more representative of the general population than the usual off-line studies conducted in experimental psychology. Participants were compensated 2.5 euros for their time.

#### Procedure and design

**Figure 6:**
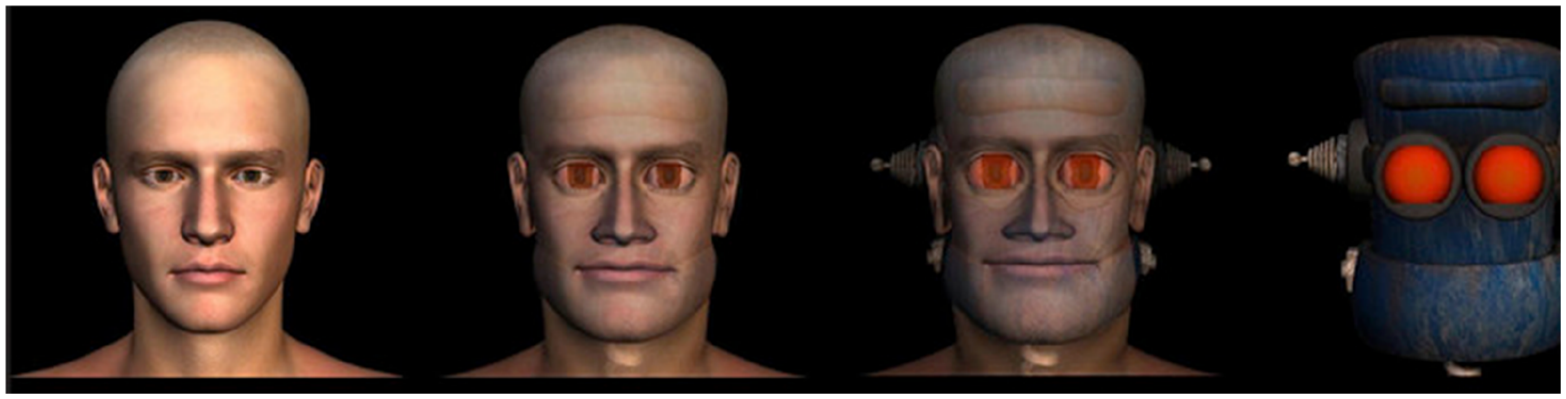
Four agents used in Study 2. From left to right: 1: CGI human; 2: Human-robot morph; 3: Robot-human morph; 4: Robot.

Our laboratory corresponded to common requirements for social psychological/cognitive science research. We used Python 2.7’s pygame library 1.96 to create a program that could not be interrupted by the participant. Participants started by giving their informed consent and were then guided to cubicles where they used headphones playing low volume pink noise to filter out potential distracting noises. Participants used a common 15.6″ laptop with a mouse. The study was a between-subjects design where participants were randomly assigned by the experimentation software in to one out of eight conditions in a 2 [Decision quality: Utilitarian vs. Deontological] × 4 [Decider: (1) CGI human, (2) human–robot morph (closer to human), (3) robot-human morph (closer to robot), and (4) robot] factorial design. The images were adapted directly from Ferrey et al. (2015; see Figure 6 for images of agents). The study design basically corresponded to a double blind experiment – neither experimenters nor the subjects knew to which condition they were randomized to.

##### Dependent variable

Participants evaluated four trolley-dilemma type vignettes in random order, with the agent shown on the left side of the dilemmas reported in Study 1. Participants indicated below each vignette how moral they found the agent’s decision to be on a Likert scale from 1 (“Very Immoral”) to 7 (“Very Moral”). All four items were averaged together resulting in a “perceived decision morality” scale with good internal consistency (Cronbach’s *α*: 0.75; *M* = 4.16, *SD* = 1.46); see also Laakasuo and Sundvall (2016) for further details on the dilemmas.

### Results and discussion

We calculated the quadratic contrast “[CGI Human + Robot] vs. [human-robot morph (closer to human) + robot-human morph (closer to robot)]” for our DV, which was not statistically significant:(*F*(1, 213) = 0.17, *p =* n.s., B = 0.14, 95% CI [−0.53, 0.82]); and we ran a two-way ANOVA for the both factors (Decider and Decision). There were no statistically significant effects.

Study 2 showed that the mere symbolic representation of the agent as either a human, a robot, or a mixture of both, is not enough to elicit the Moral Uncanny Valley effect. The results of Study 2 were also, at least partially, as expected based on previous findings since the stimulus materials were not photorealistic and did not elicit the Uncanny Valley Effect to begin with (Palomäki et al., 2018). Furthermore, both Studies (1 and 2) in unison highlight an important factor: it is important that the stimulus-materials fulfill certain criteria. Based on discussions with other researchers in this field (unpublished data), it seems that artistic depictions of robots do not work, but photorealistic materials do (Malle, 2023 – Personal communication).

## General discussion

We found a Moral Uncanny Valley effect in utilitarian decisions of High Conflict Moral Dilemmas (Study 1). This means that the appearance of the decision-making agent influences how their moral decisions are evaluated when the whole body is seen. In Study 1, utilitarian decisions were less condemned when the decision-maker was either a human or a non-creepy looking robot (Robot 2). Our exploratory analysis further showed that, when initial expectations are taken into account, the creepiest robot (Robot 4: Ishiguro) is the only one that does not seem to morally surprise or disappoint our participants when it makes the utilitarian decision (Figure 5). We also found that when the robots are creepy (Robot 3 and Robot 4), there is the highest moral approval for their decisions when they do nothing or make deontological decisions. We observed the highest difference between the expected morality of the decision-maker (measured before the experiment) and the decision, when the agents are creepy in deontological decisions. This effect also had a valley – or a mountain – shape (see Figure 5). The effect found in Study 1 aligns with previous theorizing and research (see Laakasuo et al., 2021c) but does not entirely conform to earlier findings (Malle et al., 2015, 2016; Laakasuo et al., 2021a,b,c,d,e; Zhang et al., 2022).

Unexpectedly, in addition to the uncanny valley effect found in utilitarian decisions, we also discovered a valley-shaped or “mountain-shaped” effect in the opposite direction in deontological decisions; which initially masked our main effect of Agents in Study 1. This effect was not predicted in our preregistration, and has not been reported anywhere previously. However, this effect is driven by, but not solely due to, Robot 3, which is not the creepiest robot. When we consider how moral people initially find each robot and analyze the data on the perceived morality of their decisions, we find a unique valley-shaped effect specifically for both creepy robots (Robot 3 and Robot 4: Ishiguro). It seems, that people do not expect robots, if they are not perceived as moral, to make deontological decisions in high conflict moral dilemmas.

Malle et al. (2016) showed that in two out of three studies, there was a Human-Robot asymmetry in blame attributions, but only when people make judgments about a mechanical-looking robot, not about a humanoid-looking robot; specifically robots were blamed more for the deontological inaction decisions than for utilitarian decisions. They also reported that the patterns of blame for humanoid robots were very similar to those for human agents. Zhang et al. (2022) on the other hand -- who did not use a variety of robots in their studies – do report that robots are expected to be more utilitarian in high-conflict moral dilemmas than humans. Furthermore, Laakasuo et al. (2021d) reported that the humanoid-looking robot’s decisions are found less moral overall if they are at the bottom of the uncanny valley, irrespective of the decision they make. The Study 1 shown here contradicts and supplements all of these three studies to some extent.

In Study 1, the decisions of the most humanoid robot (Robot 4: Ishiguro) and a very mechanical-looking robot (Robot 3) — both of which were statistically higher in creepiness than Robots 1 and 2 — were found to be less moral than that of Robot 2 and Human in third-degree and quadratic contrast comparisons when the decision was the action/utilitarian decision. The crucial differences here in comparison to Malle et al. (2016) are as follows: (1) They used a very different dependent variable (blameworthiness of the agent vs. morality of the decision); (2) They used a version of the basic trolley dilemma (Greene et al., 2008; Supplementary materials), which is of lower intensity than our dilemmas (impersonal vs. high conflict); (3) They used drawings produced by artists (while we used pretested stimulus materials which were actual photographs of real robots and a real human).

The differences between this paper and Laakasuo et al. (2021d) probably stem from the fact that here we use whole body stimuli and Laakasuo et al. (2021d) use only facial stimuli. Furthermore, Laakasuo et al. (2021d) did not use mediating variables in their analysis, nor did they take into consideration the baseline of the perceived morality of the agent. Also, neither of the samples in Laakasuo et al. (2021d) were as big as the sample in our Study 1. It is possible that a similar effect in their data is not visible due to lower power. With respect to Zhang et al. (2022) there are major differences, all of their samples were smaller than what we have here, and they did not measure the perceived morality of the decision. They mostly focused on how human-like the decision-maker is and how utilitarian their decisions are perceived to be, which makes comparisons between our and their findings quite challenging.

Now turning back to studies by Malle et al. (2016) and the topic of blameworthiness. Although blameworthiness is about the decision-maker and perceived morality is about decision, these dependent variables seem to align quite often in the results they produce (Malle, 2021). Therefore, it seems unlikely that the differences, where the effects of deontology and utilitarianism are essentially reversed between Malle et al. (2015, 2016) and our study, would be due to the dependent variable. It seems more likely that the most significant explanation for the differences comes from the fact that in our study, we have High Conflict Moral Dilemmas. In high-conflict moral dilemmas, the decision-maker is personally directly involved with the situation, compared to impersonal dilemmas, where there is a mediator between the decision-maker and the victims. This interpretation is supported by findings of Zhang et al. (2022).

When inspecting the differences between Study 1 and Study 2, the findings partially align with previous research, but also differ to some extent. As reported by Palomäki et al. (2018), the Uncanny Valley phenomenon is stronger with facial stimuli than with bodily stimuli, but only if the endpoints of the Uncanny Valley continuum are photorealistic. In this sense, the findings of Study 1 and Study 2 align with previous research. Nonetheless, if we use whole body imagery, the moral judgments of robot decisions seem to follow the Moral Uncanny Valley effects to some extent, but not perfectly. Furthermore, if we use stimuli that do not reliably elicit the Uncanny Valley effect, even if it is facial stimuli, the effect does not emerge simply by using symbolic or CGI-based stimuli. It seems that human perceptions regarding the agent’s appearance and how it influences the evaluation of their decisions are complex and contingent on the quality of the stimulus materials.

Both of these studies suffer from various limitations, standard in this type of research. The participants are potentially susceptible to different sorts of demand characteristics (i.e., they try to provide the researchers with the data they think they need). They are never fully representative of the whole population and are probably more open-minded and curious than the population average since they did decide to participate in such studies. Furthermore, studies in this area suffer from the virtual non-use of standardized stimulus materials built with scientific consensus that would present robots reliably on the “human-likeness” continuum.^5^ What human-likeness means in such contexts is not entirely clear either. Thus, the results from these two studies should be read and interpreted with caution, as they do not perfectly align with any deeply understood theories in either moral judgment formation (Malle et al., 2021) or the Uncanny Valley Effect (Diel et al., 2021). Similarly, there is no scientific consensus on which dependent variables should be used and whether we should be evaluating the decision-maker (e.g., Malle et al., 2015, 2016; Bigman and Gray, 2018) or the decisions and their consequences (Laakasuo et al., 2023; Sundvall et al., 2023). Furthermore, the results presented here are not super highly powered and are one-off studies that would need to be replicated independently by other research groups, particularly Study 1. Nonetheless, the results seem to fit with the existing studies, which mitigates these concerns.

Despite the limitations in these studies, the findings presented here do deepen our understanding of the moral judgment research with respect to decisions made by robots. Firstly, there is some indication here that it is not necessarily about how mechanical or human-like the robot looks like and it is not about the decision itself, but rather about how much the robot’s appearance aligns with our initial expectations of their future behavior. This finding, though requiring further confirmation, introduces an intriguing nuance to our understanding of moral judgment of robotic and – more controversially – of human agents and their decisions.

Secondly, we observed that the evaluation of decisions made by robots might depend on the type of decision (utilitarian vs. deontological) and the perceived creepiness of the robot. The impact of these factors appears to be more significant than initially expected, suggesting a need to further explore the complex interplay between the type of moral decision, the agent’s physical appearance, and the observers’ initial expectations and perceptions (as suggested by Sullivan and Wamba, 2022).

Thirdly, it seems that, contrary to previous assumptions, the Uncanny Valley Effect is not simply driven by a discrepancy between an agent’s appearance and how much mind they are perceived to have (see exploratory analyses). Instead, our results suggest that the Moral Uncanny Valley might also arise from a discrepancy between an agent’s appearance and the moral expectations that people have about them. This point is important for moving forward in the field of moral psychology of AI, robotics and transhumanism (see Laakasuo et al., 2018; Laakasuo et al., 2021e; Koverola et al., 2022b). This discrepancy might be particularly important when the decision at stake is a high conflict moral dilemma, where the decision-maker is personally directly involved with the situation. Future studies should separate robot moral decisions into high conflict, impersonal and low conflict equivalents and take into consideration how people feel about robots before people see them interacting in morally ambiguous situations (Zhang et al., 2022; see Koverola et al., 2022a for a recent instrument).

Moreover, it appears that the Uncanny Valley might be more or less prominent depending on the nature of the stimuli and whether they elicit the Uncanny Valley Effect to begin with. This means that symbolic or CGI-based stimuli may not be sufficient to elicit the Uncanny Valley, and it further highlights the importance of realistic, high-quality stimuli in research related to the Uncanny Valley and moral judgment of robotic agents.

Lastly, our findings indicate a potential interaction between the Uncanny Valley and moral judgment, which was not predicted in our pre-registration and has not been reported anywhere previously. It seems to matter who is it that stays inactive in high-stakes situations. This novel finding suggests a need for further research and opens up a new avenue of investigation in the moral judgment field.

While this study contributes to our understanding of the moral judgment of robotic agents and the Uncanny Valley, it also highlights the complexity of this field and the existence of many factors at play. The lack of standardized materials, the difficulty in reliably presenting robots on the human-likeness continuum, and the varied interpretations of human-likeness in this context are all challenges that future research will need to address (Phillips et al., 2023). Furthermore, it underscores the need for careful interpretation of results and consideration of the effects of the chosen dependent variables. Therefore, we need more volume of research in this area and peer review practices that give leeway innovative study designs.

Despite these challenges, we believe that our study offers valuable insights and lays the groundwork for future investigations in this field. Our findings underline the need for a nuanced understanding of the Uncanny Valley and its impact on moral judgment, as well as the necessity for further research to explore the complex interplay between robot appearance, type of decision, moral expectations and moral judgment. Ultimately, we hope that our research will contribute to the development of more effective and ethically aware robotic systems.

## Conclusion

To summarize, we successfully ran two studies, that strengthened and refined and problematized previous findings in moral psychology of robotics. We showed that the Moral Uncanny Valley can be reproduced with another stimulus set; we showed that simple representations of humans and robots are not enough to produce this effect and we showed that the initial moral expectations of the decision-making agent might play a key role in understanding how people will feel about future robots that will be joining us in this society sometime sooner rather than later. This type of research is crucial for human well-being in the future.

## Data availability statement

Data will be available at doi: 10.6084/m9.figshare.24417127 upon the publication of this article and once it has been anonymized according to the requirements of GDPR. The data was collected before GDPR was implemented and therefore requires extra management efforts.

## Ethics statement

The requirement of ethical approval was waived by University of Helsinki Ethical Review Board in Humanities and Social and Behavioral sciences for the studies involving humans because according to the guidelines of the Finnish National Board on Research Integrity, this type of research does not require ethical review (https://www.tenk.fi/en). The studies were conducted in accordance with the local legislation and institutional requirements. The participants provided their written informed consent to participate in this study.

## Author contributions

ML: Conceptualization, Formal analysis, Funding acquisition, Investigation, Methodology, Project administration, Resources, Supervision, Visualization, Writing – original draft, Writing – review & editing.

## Footnotes

^1^Why the asymmetry effect exists and what would explain it, is not currently known, but several authors have raised the issue recently (Laakasuo et al., 2021a,^2023^;Stuart and Kneer, 2021;Sundvall et al., 2023).

^2^ osf.io/ukwdz

^3^ www.prolific.co

^4^After this we did the same analysis with Trust and Likability, but the results were not essentially different.

^5^ https://www.abotdatabase.info/
