# Supplementary material for: Moral Uncanny Valley revisited – how human expectations of robot morality based on robot appearance moderate the perceived morality of robot decisions in high conflict moral dilemmas
Source: Front Psychol. 2023 Nov 21;14:1270371. doi: 10.3389/fpsyg.2023.1270371 (PMC10706005; doi:10.3389/fpsyg.2023.1270371)
Supplement: Supplementary file 1 [file Data_Sheet_1.PDF]

APPENDIX

List of Moral Dilemmas used in the Studies

(Adapted from Greene et al. 2008); Laakasuo & Sundvall, 2016)

1. Mountaineering

The agent is the leader of a mountaineering expedition that is stranded in the wilderness. The expedition includes a family of six that has a genetically caused vitamin deficiency. A few people's kidneys contain large amounts of this vitamin.

There is one such person in the group. The only way to save the lives of the six members of this family is to remove one of this man's kidneys so that the necessary vitamins may be extracted from it. The man will not die, but his health will be compromised. The man is opposed to this plan, but the agent has the power to decide anyways.

[PAGE BREAK]

Utilitarian: The agent decides to remove the kidney.

Deontological:The agent decides not to remove the kidney.

How moral do you find the agents decision?

1 = very immoral – 7 = very moral

2. Submarine

The agent is the captain of a military submarine traveling underneath a large iceberg. An on-board explosion has caused the agent to lose most of the oxygen supply and has injured one of the crew who is quickly losing blood. The injured crew member is going to die from his wounds no matter what happens.

The remaining oxygen is not sufficient for the entire crew to make it to the surface. The only way to save the other crew members is to shoot dead the injured crew member so that there will be just enough oxygen for the rest of the crew to survive.

[PAGE BREAK]

The agent decides to kill the fatally injured crew member.

The agent decides not to kill the fatally injured crew member.

How moral do you find the agents decision?

1 = very immoral – 7 = very moral

3. Terrorist

The agent is negotiating with a powerful and determined terrorist who is about to set off a bomb in a crowded area. The agent's one advantage is that the agent has his teen-age son in custody.

There is only one thing that the agent can do to stop the terrorist from detonating the bomb, which will kill

thousands of people if detonated. To stop him, the agent must contact him over the satellite hook-up that he has established and, in front of the camera, break one of his son's arms and then threaten to break the other one if he does not give himself up.

[PAGE BREAK]

The agent decided to break the terrorist's son's arm.

The agent decided (not) to break the terrorist's son's arm.

How moral do you find the agents decision?

1 = very immoral – 7 = very moral

4. Footbridge

A runaway trolley is heading down the tracks toward five workmen who will be killed if the trolley proceeds on its present course. The agent is on a footbridge over the tracks, in between the approaching trolley and the five workmen. Next to the agent on this footbridge is a stranger who happens to be very large.

The only way to save the lives of the five workmen is to push this stranger off the bridge and onto the tracks below where his large body will stop the trolley. The stranger will die if the agent does this, but the five workmen will be saved.

[PAGE BREAK]

The agent decided to push the stranger on to the tracks

The agent decided not to push the stranger on to the tracks

How moral do you find the agents decision?

1 = very immoral – 7 = very moral

5. Euthanasia

The agent is the leader of a small group of soldiers. The group is on its way back from a completed mission deep in enemy territory when one of the men stepped into a trap that has been set by the enemy and is badly injured. The trap is connected to a radio device that by now has alerted the enemy to the group's presence. They will soon be on their way.

If the enemy finds the injured man they will torture him and kill him. He begs the agent not to leave him behind, but if the agent tries to take him along the entire group will be captured. The only way to prevent this injured soldier from being tortured is to shoot him.

[PAGE BREAK]

How moral do you find the agents decision?

1 = very immoral – 7 = very moral
